# A Triple-Transgenic Immunotolerant Mouse Model

**DOI:** 10.1002/jps.23447

**Published:** 2013-01-11

**Authors:** Nina Brenden, Katja Madeyski-Bengtson, Klara Martinsson, Rebecka Svärd, Sara Albery-Larsdotter, Britta Granath, Hanna Lundgren, Ann Lövgren

**Affiliations:** 1 AstraZeneca, Safety AssessmentSödertälje, Sweden; 2 AstraZeneca, Discovery SciencesMölndal, Sweden; 3 AstraZeneca, Innovative MedicinesMölndal, Sweden

**Keywords:** bioequivalence, pharmacokinetics/pharmacodynamics, protein formulation, glycoprotein, FII, FVII, FX, immunogenicity, transgenics

## Abstract

Avoiding unwanted immunogenicity is of key importance in the development of therapeutic drug proteins. Animal models are of less predictive value because most of the drug proteins are recognized as foreign proteins. However, different methods have been developed to obtain immunotolerant animal models. So far, the immunotolerant animal models have been developed to assess one protein at a time and are not suitable for the assessment of combination products. Our aim was to develop an animal model for evaluating the impact of manufacturing and formulation changes on immunogenicity, suitable for both single protein and combination products. We constructed two lines of transgenic mice expressing the three human coagulation factors, II, VII, and X, by inserting a single vector containing the three coagulation factors encoding sequences separated by insulator sequences derived from the chicken beta-globin locus into the mouse genome. Immunization of transgenic mice from the two lines and their wild-type littermates showed that transgenic mice from both lines were immunotolerant to the expressed human coagulation factors. We conclude that transgenic mice immunotolerant to multiple proteins can be obtained, and that these mice are potentially useful as animal models in the assessment of immunogenicity in response to manufacturing changes. © 2013 Wiley Periodicals, Inc. and the American Pharmacists Association J Pharm Sci 102:1116–1124, 2013

## INTRODUCTION

During the development of therapeutic proteins, one of the most important aspects that needs to be addressed is their potential immunogenicity. Treatment with therapeutic proteins having a nonredundant endogenous counterpart could potentially trigger an immunological response leading to neutralization of the endogenous proteins and adverse events. Predicting risks for immunogenicity is often difficult as therapeutic proteins are very complex molecules. Because of the different immune mechanisms, the value of animals as predictive models is limited. A human protein administered to animals will evoke a classical immune response because of the exogenous character of the protein, whereas patients treated with the same protein will evoke a different immune response. Coagulation factor concentrates containing one or more proteins are important therapeutic proteins in the treatment and prevention of bleeding, and the use of such concentrates are known to, at varying frequency, trigger formation of antidrug antibodies that may also be neutralizing (inhibitor formation). Inhibitor formation may occur both from the treatment with plasma-derived and recombinant concentrates of FVIII or FIX, but inhibitors to FVII are very unusual.^1^,^2^ Formation of neutralizing antibodies may have serious consequences as in the case with recombinant erythropoietin, where neutralizing antibodies arose because of the changes in the formulation and caused pure red cell aplasia.^3^ To avoid unnecessary immunogenicity associated with manufacturing and formulation processes, transgenic animal models could be of value to study the assessment of relative immunogenicity between products and differences in response to drug delivery, such as a route of administration, dosage, and frequencies as relative immunogenicity would presumably be similar between humans and nonhuman models.^4^

Immunotolerant animal models can be used to study breaking of B-cell tolerance and drug-induced antibody formation.^5^ Immunotolerance can be obtained by neonatal tolerance induction or by using transgenic mice expressing human proteins.^6^,^7^ Tolerance to FVIII has been induced by both intraperitoneal and intravenous injections of recombinant human FVIII to neonatal mice within 30 h after birth. Treated mice were found to be tolerant to subsequent injections of rFVIII in adult life, and the tolerance was antigen specific.^8^,^9^ Neonatal tolerance induction has also been induced in rats using recombinant human FVII.^10^ Tolerance models using transgenic mice have been developed for human IFNα, IFNβ, and FVIII, among others.^5^,^11^–^13^ All of the models show tolerance to the native human protein, whereas modifications of the protein, for example, aggregation, had an impact on immunogenicity. Thus transgenic mice offer an opportunity to study the mechanism of tolerance against self-proteins as well as breakage of tolerance.^7^,^14^ This has been demonstrated in several models with proteins like INFα, FVIII, and IFNβ.^5^,^11^,^13^,^15^

In this paper, we present the development of a unique triple-transgenic model, in which sequences encoding the three human coagulation factors II (prothrombin), VII, and X have been inserted into the mouse genome making the mice triple transgenic, expressing all three human coagulation factors. This triple-transgenic model has been tested in relative immunogenicity studies using a combination of recombinant human FII, FVII, and FX.

## MATERIAL AND METHODS

### Animal Ethics

All animal experiments described in this paper have been approved by the Animal Ethics Committee of Gothenburg, Sweden.

### Construction of Transgenic Mouse Strains

**Figure 1:**
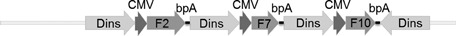
Outline of the triple factor DNA construct injected into fertilized eggs.

We have generated triple transgenic mice, which express the human coagulation factors II, VII, and X, under the control of the cytomegalovirus (CMV) promoter. The vector was designed to harbor all three factors in tandem, although as separate expression units. To minimize dysregulation and/or integration effects, each expression unit was flanked by double 1.2 kb insulator including CCCTC-Binding Factor (CTCF) binding sites, derived from the chicken beta-globin locus.^16^ A Kozac sequence, GCCGCCACC, was placed −1 nt upstream of the ATG start codon of each cDNA, and to stabilize the transcript, the bovine growth hormone polyadenylation signal (bGH polyA) was attached to the 3′ end of each transcription unit (Fig. 1). The resulting transgenic DNA construct was injected into the pronucleus of fertilized C57Bl/6NCrl (Charles River Laboratories, Sulzfeld, Germany) eggs and implanted into pseudo pregnant foster mothers. Eight founder lines, A–H, were derived and two of them, Line E; C57Bl/6-Tg(F2, F7, F10)^EAztc^, and Line H; C57Bl/6-Tg(F2, F7, F10)^HAztc^, proved to express all of the three human coagulation factors. Genotypes were determined by PCR amplification of genomic DNA, derived from mouse ear biopsies, using primers CMV F1; 5′-GTAGGCGTGTACGGTGGGAG-3′, F2 Rev1; 5′-GCCCAGAACACATCCGTAGC-3′, F7 Rev1; 5′-AGCACTGCTCCTCCTTGCAC-3′, and F10 Rev1; 5′-CGTGGCAGAACTGGTCACAG-3′, in the same PCR reaction giving rise to the following band sizes: FII; 306 bp, FVII; 233 bp, and FX; 476 bp.

### Verification of Human Coagulation Factor Expression

Transgenic mice were identified by genotyping using PCR assays to detect sequences encoding human FII, FVII, and FX. Expression of the human coagulation factors was tested by three different ELISA assays specific for human FII, FVII, and FX, respectively. EDTA plasma samples were prepared from transgenic mice and wild-type littermates approximately 12 weeks of age. Plasma samples were stored frozen at −70°C or colder until analysis. Nunc Maxisorp plates were coated with 100 µL/well monoclonal antibody directed to human FII (0.33–2 µg/mL, product P9115-04, Nordic Biosite, Täby, Sweden), human FVII (2 µg/mL product 2282, American Diagnostica, Pfungstadt, Germany) or human FX (2 µg/mL product GMA-520, Green Mountain Antibodies, Burlington, Vermomt), diluted in carbonate coating buffer (product C3041, Sigma Aldrich, Stockholm, Sweden). Plates were coated overnight on a refrigerated shaker or wobbling table and then blocked with 100 µL/well phosphate-buffered saline (PBS) containing 1% BSA (PBS/BSA) for 1 h at room temperature. Plates were washed three times with PBS containing 0.05% (w/v) TWEEN 20 using a plate washer. Samples were thawed at room temperature and diluted five times for the detection of human FII and 10 times for the detection of human FVII or human FX in PBS/BSA. Standard curves were prepared in PBS/BSA containing 10% pooled EDTA plasma from wild-type mice (FVII and FX) or 20% pooled EDTA plasma from wild-type mice (FII). Standard range for human FII was 5–250 ng/mL, for human FVII 2–64 ng/mL, and for human FX 5–160 ng/mL. After addition of 50 µL/well sample or standard, plates were incubated on a wobbling table at room temperature for 1.5 h and washed as before. Detection antibody diluted in PBS/BSA was added at 50 µL/well. FII detection antibody; ab9020 (Abcam, Cambridge, UK) was diluted 1:2000–4000, FVII detection antibody; SAFVII-AP (Enzyme Research Laboratories, South Bend, Indiana) was diluted 1:4000, and FX detection antibody; SAFX-AP (Enzyme Research Laboratories) was diluted 1:2000. Plates were incubated for 1 h on a wobbling table at room temperature and washed as before. Mouse α-Sheep/Goat IgG conjugated to alkaline phosphatase (Sigma Aldrich A8062) was diluted 1/4000 in PBS/BSA, 50 µL was added to wells and plates were incubated on a wobbling table at room temperature for 1 h. After washing of the plates, as before, 50 µL/well of p-NPP solution (p-nitrophenyl phosphate, Sigma Aldrich N27709) was added. After suitable formation of yellow color, the reaction was stopped by addition of 20 µL/well 2 M sodium hydroxide. Absorbance was read at 405 nm using Softmax software. Transgenic mice from two lines, E and H, were judged to produce detectable amounts of human FII, FVII, and FX.

### Mouse Immunization

Transgenic mice, approximately 12 weeks of age, from lines E and H, were used in an immunization study and wild-type littermates were included as controls. Six wild-type and six transgenic mice from each line and gender were included and dosed with 1 mg/kg, each of recombinant human FII, FVII, and FX. The mice were given a single subcutaneous injection just below the neck every second week with a total of four subcutaneous injections. The chosen dose level and administration route were based on previous knowledge on requirements for induction of antibody production to autologous proteins using transgenic mice.^17^ Plasma samples were taken predose and two weeks after the last injection, and kept frozen at −70°C until analysis.

### Compound and Formulation

The mice were injected with a combination of 1 mg/kg of each recombinant human FII, FVII, and FX. The protein solution was prepared in a 2 mM trisodium citrate, 10 mM histidine, and 140 mM NaCl, pH 7.4 buffer containing <0.01 EU/mL endotoxin. The concentration of each test compound was determined by UV spectroscopy, and the purity was assessed by size-exclusion chromatography. To assure bioactivity, the potency of the proteins was tested by coagulation factor assays for FII, FVII, and FX. FII potency was tested by a prothrombinase assay (Kirchhof et al.,^18^ commercially available as Rox Prothrombin, Art No. 200040, Rossix, Mölndal, Sweden). FVII potency was tested by the Biophen FVII assay (Aniara, Mason, Ohio, cat. No. A221304), and FX potency was tested by the Biophen FX assay (Aniara, cat. No. A221705). These assays were also used to estimate the concentration of mouse coagulation factors II, FVII, and X in mouse plasma.

### Antidrug-Specific Ig Titers

Human FII-, FVII-, and FX-specific antibody titers were measured in the plasma samples using ELISA. FII, FVII, and FX were biotinylated with an EZ-Link Sulfo-NHS-LC-biotinylation kit (Pierce, Rockford, Illinois) according to vendor instruction, and the biotinylated recombinant FII (1 mg/L), FVII (5 mg/L), or FX (5 mg/L) was added to prewashed streptavidine-coated plates (Nunc, Roskilde, Denmark). The plates were incubated for 1 h at room temperature under slow shaking (FII) or overnight at 4°C (FVII and FX) and thereafter washed and blocked. The plates were washed and sample diluted in assay buffer was added to each well and incubated for 2 h at room temperature. The plates were thereafter washed, and 100 µL of polyclonal rabbit α-mouse immunoglobulin antibody conjugated with horseradish peroxidase (Dako, Copenhagen, Denmark) diluted in assay buffer was added. After 1-h (FVII and FX) or 2-h (FII) incubation, the plates were washed and EC-Blue®-enhanced substrate was added (Medicago, Uppsala, Sweden). After 5 min (FVII and FX) to 15 min (FII) at room temperature, 0.5 M HCl was added to stop the reaction, and the absorbance was read at 450 nm on a SpectraMax Plus microplate reader (Molecular Devices, Sunnyvale, California). Each plate contained one standard curve with a range between 0.1 and 20 ng/mL (FII) or 0.5 and 1000 ng/mL (FVII and FX).

A screening was first performed where all samples were diluted 1:100 in assay buffer and analyzed in triplicate. Samples showing an optical density (OD) value above the standard curve limit were further diluted and analyzed in triplicates.

### Statistical Analyses

To discriminate positive responses from background, an upper negative limit of 95% for the cut-point was used; a cutoff value from the predose samples was calculated using OD values accordingly:


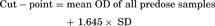


where 1.645 is the 95th percentile of the normal distribution.^19^

The cut-off was calculated to OD_450_ of 0.438 (FII), 0.103 (FVII), and 0.043 (FX), and values above this cut-off point were regarded as positive.

Differences between pre- and postdose samples were analyzed using OD values and a Paired *t*-test. A value of *p* < 0.05 was considered to be statistically significant.

Data from positive responders were generated as concentrations (ng/mL) obtained from OD values correlated to a standard curve from the ELISA analyses. Log-transformed data showed normal distribution and was used to calculate possible significant differences in antidrug-specific antibody concentrations between the different groups using one-way ANOVA. A value of *p* < 0.05 was considered to be statistically significant.

## RESULTS

We have successfully constructed triple-transgenic mice expressing three human coagulation factors by inserting a vector containing their encoding sequences and control sequences separated by beta-globin insulator sequences into the mouse genome (Fig. 1). The concentration of the three human coagulation factors in plasma samples from transgenic mice was estimated to be approximately 10–50 ng/mL and was similar for all three coagulation factors (data not shown), indicating that the use of the insulator sequences had the desired effect. The transgenic expression levels of the coagulation factors were low compared with murine endogenous levels; the murine endogenous level of FII was measured to approximately 100 µg/mL, the FVII level to approximately 1 µg/mL, and FX was measured to approximately 8 µg/mL. These murine endogenous levels are comparable to human endogenous levels (data not shown). At the achieved expression levels, the addition of the human coagulation factors is therefore not likely to affect the mouse coagulation system, and no adverse effects or alteration of phenotype was observed for the transgenic mice. To test if transgenic mice differed from wild-type mice in their ability to produce antibodies to human FII, FVII, or FX, transgenic mice and wild-type littermates were immunized with a mix of recombinant human FII, FVII, and FX, as described in “Material and Methods.” Plasma samples were collected before immunization and 2 weeks after the last administration. Samples were analyzed for antibodies to human coagulation factors, and the assay cut-point for scoring a positive response was set in accordance with regulatory recommendations (cut-point, 5% positive), as described in “Material and Methods.”^17^

In summary, there was a large difference in the development of coagulation-factor-specific antibodies between wild-type and transgenic animals following the treatment. The transgenic animals responded with lower levels of antibodies, even though significance could not be reached in some groups because of the large individual variation. For the different coagulation factors, the results are given below.

### Factor II

**Figure 2:**
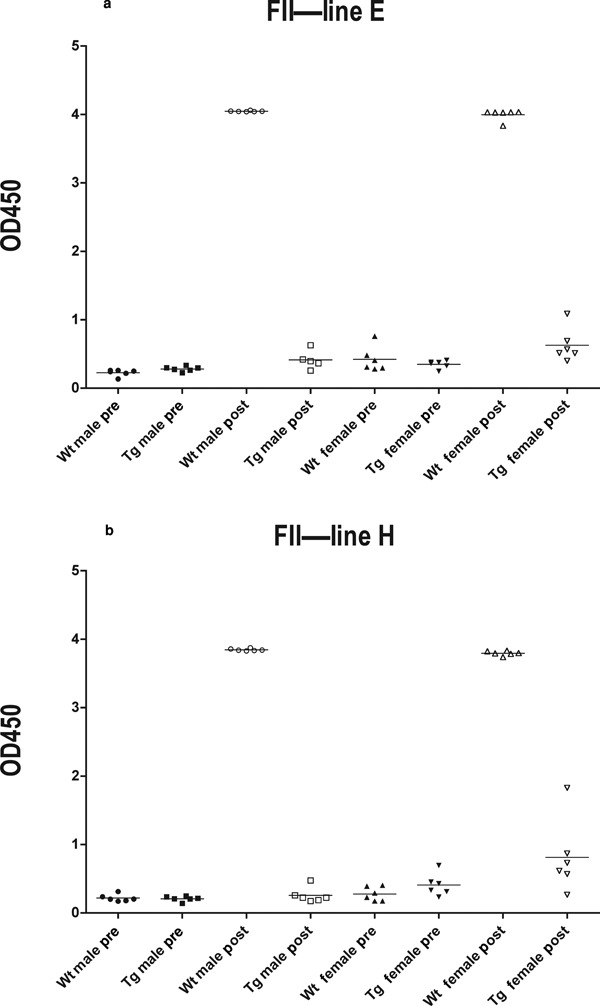
Human FII-specific antibody titers in pre- and posttreatment samples from wild-type and transgenic animals from line E (a) and line H (b), measured by ELISA and expressed as optical density (OD). Results were obtained from the screening assay where all samples were diluted 1:100. One transgenic male mouse from line E died during the study. Mean OD values are indicated by horizontal bars. Cut-off value was calculated to 0.438, values above this cutoff point were regarded as positive. Wt, wild type; Tg, transgenic.

Pre- and postdosing samples were first compared. As expected, wild-type male and female mice from both line E (*p* < 0.0001 and *p* < 0.0001, respectively) and H (*p* < 0.0001 and *p* < 0.0001, respectively) showed a significant increase in antihuman FII-specific antibodies following dosing. Transgenic male mice from line E and line H and transgenic female mice from line H did not develop any significant levels of human FII-specific antibodies. However, transgenic female mice from line E showed a significant increase in human FII-specific antibodies, but the OD values were just above the cut-point for positive responses (*p* = 0.03) (Fig. 2, Table 1).

**Table 1:** Statistical Comparison of the Specific Antibody Response Between Samples from Wild-Type (Wt) and Transgenic (Tg) Mice Before and After Treatment with a Combination of Recombinant Human FII, FVII, and FX

		FII		FVII		FX	
				
		Line E	Line H	Line E	Line H	Line E	Line H
Wt	Male	**<0.0001**	**<0.0001**	**0.0001**	**<0.0001**	0.06	**0.04**
	Female	**<0.0001**	**<0.0001**	**<0.0001**	**<0.0001**	**0.001**	**<0.0001**
Tg	Male	0.06	0.32	**0.04**	0.07	0.20	**0.003**
	Female	**0.03**	0.13	0.13	**0.0001**	**0.01**	**0.04**

Differences between pre- and postdose samples were analyzed using OD values and a Paired *t*-test. A value of *p* < 0.05 was considered to be statistically significant, marked with bold.

Group comparisons of postdose samples were done between animals with antibody levels above cut-point, and data showed that wild-type female mice from both lines responded with significantly higher levels of prothrombin-specific antibodies compared with transgenic female mice from respective line. All wild-type male mice from both lines responded with high levels of prothrombin-specific antibodies following treatment, whereas only one transgenic male mouse from each line showed prothrombin-specific antibody levels above the cut-off (Table 2).

**Table 2:** Statistical Comparison of Values Above Cut-Point, Between Genders Within Respective Line, and Between Wild-Type (Wt) and Transgenic (Tg) Mice Following a Combination Treatment of Recombinant Human FII, FVII, and FX

Group 1	Group 2	*p* value
FII		
Line E Wt female	Line E Tg female	*p* =*0.001*
Line H Wt female	Line H Tg female	*p* = 0.001
FVII		
Line E Wt male	Line E Tg male	*p* = 0.001
Line E Wt female	Line E Tg female	*p* = 0.001
Line H Wt male	Line H Tg male	*p* = 0.001
Line H Wt female	Line H Tg female	*p* = 0.001
Line E Wt male	Line E Wt female	*p* = 0.01
Line E Tg male	Line E Tg female	*p* = 0.05
Line H Wt male	Line H Wt female	*p* = 0.001
FX		
Line E Wt female	Line E Tg female	*p* = 0.01
Line H Wt male	Line H Tg male	*p* = 0.05
Line H Wt female	Line H Tg female	*p* = 0.001
Line H Wt male	Line H Wt female	*p* = 0.05

Log-transformed data were used to calculate significant differences in antidrug-specific Ig concentrations between the different groups using one-way ANOVA.

### Factor VII

**Figure 3:**
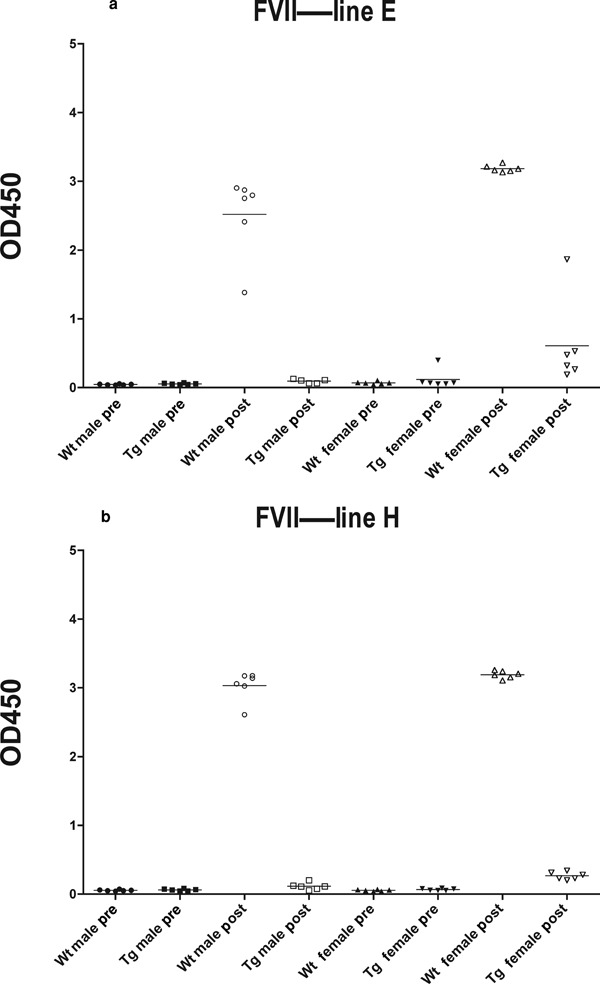
Human FVII-specific antibody titers in pre- and posttreatment samples from wild-type and transgenic animals from line E (a) and line H (b), measured by ELISA and expressed as OD. Results were obtained from the screening assay where all samples were diluted 1:100. One transgenic male mouse from line E died during the study. Mean OD values are indicated by horizontal bars. Cut-off value was calculated to 0.103, values above this cutoff point were regarded as positive. Wt, wild type; Tg, transgenic.

All wild-type mice showed a high, significant increase in anti-human FVII-specific antibodies when pre- and postdosing samples were compared (E: *p* = 0.0001, *p* < 0.0001 H: *p* < 0.0001, *p* < 0.0001). Transgenic male mice from line E (*p* = 0.04) and transgenic female mice from line H (*p* = 0.0001) also showed a significant increase in drug-specific antibodies when comparing pre- and postdose samples, but the responses were very low, and the OD values were just above the cut-point for positive responses. Transgenic male mice from line H and transgenic female mice from line E showed a small increase in FVII-specific antibodies, but because of the large individual variation, there was no significant difference (Fig. 3, Table 1).

When running a group comparison between groups with antibody values above cut-point, wild-type male, and female mice from both line E and H, as expected, showed significant higher levels of FVII-specific antibodies compared with respective transgenic strain posttreatment. Wild-type female mice from both lines and transgenic female mice from line E showed a significantly higher level of FVII-specific antibodies compared with respective male mice posttreatment (Table 2).

### Factor X

**Figure 4:**
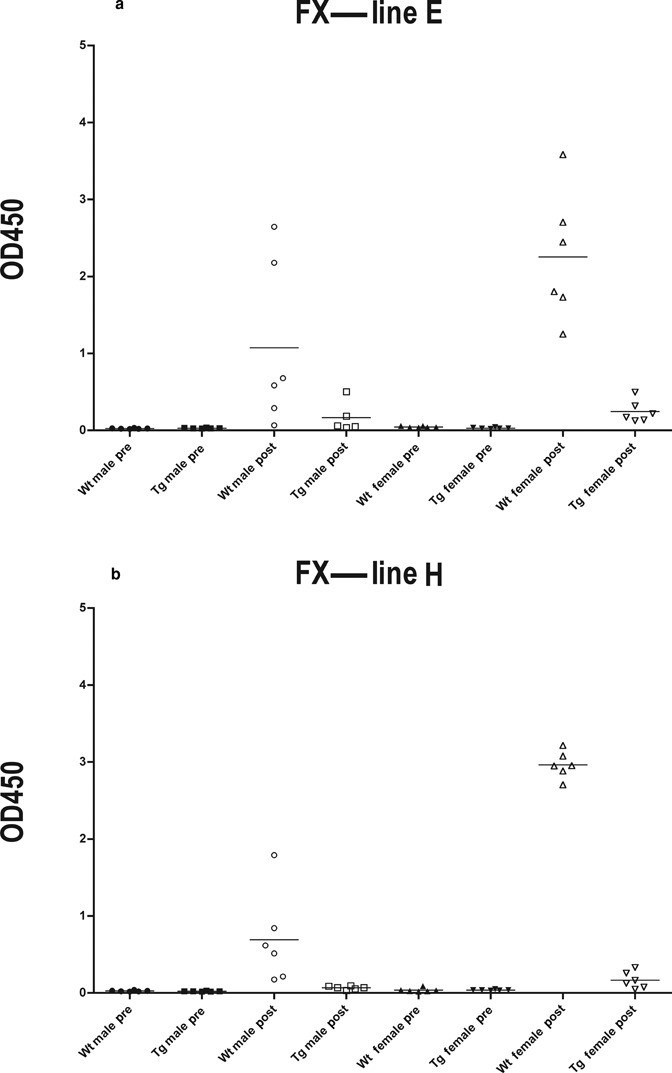
Human FX-specific antibody titers in pre- and posttreatment samples from wild type and transgenic animals from line E (a) and line H (b), measured by ELISA and expressed as optical density (OD). Results were obtained from the screening assay where all samples were diluted 1:100. One transgenic male mouse from line E died during the study. Mean OD values are indicated by horizontal bars. Cut-off value was calculated to 0.043, values above this cutoff point were regarded as positive. Wt, wild type; Tg, transgenic.

Wild-type male mice from line H (*p* = 0.04) and female mice from both line E and H (E: *p* = 0.001 H: *p* < 0.0001) showed a significant increase in FX-specific antibodies following dosing. The OD values for the FX antibodies showed a higher degree of variability among the individuals compared with the prothrombin- and FVII-specific antibodies. Because of this variation, the response from wild-type male mice from line E did not show a significant response when pre- and postdose samples were compared. Transgenic female mice from line E (*p* = 0.01) and transgenic male and female mice from line H (*p* = 0.003 and *p* = 0.04, respectively) showed a significant increase in FX-specific antibodies following dosing. However, the OD values were just above the cut-point for positive responses, and the biological significance is unclear (Fig. 4, Table 1).

Group comparisons between animals with responses above cut-point showed that wild-type female mice from both lines and wild-type male mice from line H, but not to line E because of large variation, showed a significantly higher level of FX-specific antibodies compared with respective transgenic strain posttreatment. Wild-type female mice from line H showed significantly higher levels of FX-specific antibodies than wild-type male mice from line H and wild-type female mice from line E (Table 2).

## DISCUSSION

Today, it is not possible to predict immunogenicity before entering clinical trials because it is difficult to identify all factors responsible for induction of immunogenicity.^5^ Thus, better preclinical models are required and transgenic models could be one such model system. Use of transgenic mouse model systems could, for instance, help evaluate different drug modifications, as has been shown by van Helden et al,^20^ where hFVIII transgenic mice were tolerant to the native human protein but were able to mount an antibody response to different modifications of the protein. Similar models could be useful to identify modifications that potentially break immune tolerance to the protein, as suggested by others.^21^

Our work was performed to generate a transgenic mouse model to study immunological responses to human FII, FVII, and FX, given as single factors or in combination. By designing a construct that harbors all three coagulation factors, FII, FVII, and FX, in tandem, although as separate expression units, these immunological studies could be run using one single mouse model. Another advantage of having the factors linked to each other in equimolar amounts is that they will integrate into the same locus of the mouse genome, which increases the likelihood of even expression levels. The breeding efficiency also improves, as segregation of the factors is prevented, which dramatically reduces the numbers of animals needed and simplifies genotyping of offspring as one PCR reaction can amplify fragments from all three coagulation factor sequences simultaneously.

The two transgenic mouse strains obtained responded similarly when challenged with the human coagulation factors II, VII, and X and may both be useful in future studies. Although the obtained expression level was low judged by the amounts of human coagulation factors detected in plasma samples, it was sufficient to induce tolerance to human coagulation factors II, VII, and X. However, the CMV promoter used to control human FII, FVII, and FX expression in the mice is likely to generate expression in many tissues, which can contribute to inducing tolerance.

Coagulation factors are well conserved among animal species and are often poorly immunogenic if administered intravenously. We therefore chose the subcutaneous route of administration as this administration route is known to increase potential immunogenicity, and less number of administrations would be required. Wild-type mice responded strongly with drug-specific antibodies following subcutaneous injections with human FII, FVII, and FX. The transgenic mouse strains produced very low titers of drug-specific antibodies, just above the assay cut-point, demonstrating that a high degree of tolerance to the human coagulation factors had been achieved. Complete tolerance is not desired if the mouse strain should be used to detect changes in immunogenicity (i.e, as a result of manufacturing changes). We have generated a human triple-transgenic mouse model, and we have shown that the two transgenic lines are tolerant when given a combination of recombinant human FII, FVII, and FX. However, whether break of tolerance is achieved when injecting modified versions of these coagulations factors, as a result of modifications during manufacturing or because of significant glycosylation changes, has not yet been demonstrated. Future studies of interest would be to test the tolerance of these transgenic strains using, for instance, different drug products derived from different manufacturing processes.

In conclusion, we believe that transgenic mice may be a valuable tool in assessing the risk of immunogenicity of a product, although results from animal models are difficult to translate to humans and immunogenicity cannot be fully excluded. The successful construction of mice expressing three different proteins shows that combination products can also be evaluated in immunotolerant animals. However, one model cannot stand alone, and a combination of experimental systems is required, involving not only *in vivo* models but also advanced analytical characterizations,^22^ and perhaps this could help to improve the understanding of the mechanisms behind immunogenicity and improve risk mitigation when entering the clinic.
